# Application of Urine-Derived Stem Cells to Cellular Modeling in Neuromuscular and Neurodegenerative Diseases

**DOI:** 10.3389/fnmol.2019.00297

**Published:** 2019-12-05

**Authors:** Mitsuto Sato, Hotake Takizawa, Akinori Nakamura, Bradley J. Turner, Fazel Shabanpoor, Yoshitsugu Aoki

**Affiliations:** ^1^Department of Molecular Therapy, National Institute of Neuroscience, National Center of Neurology and Psychiatry, Kodaira, Japan; ^2^Department of Medicine (Neurology and Rheumatology), Shinshu University School of Medicine, Matsumoto, Japan; ^3^Department of Clinical Research, National Hospital Organization Matsumoto Medical Center, Matsumoto, Japan; ^4^The Florey Institute of Neuroscience and Mental Health, University of Melbourne, Parkville, VIC, Australia

**Keywords:** urine-derived stem cells (USCs), induced pluripotent stem cells (iPSCs), disease modeling, direct-reprogramming, precision medicine

## Abstract

Neuromuscular and neurodegenerative diseases are mostly modeled using genetically modified animals such as mice. However, animal models do not recapitulate all the phenotypes that are specific to human disease. This is mainly due to the genetic, anatomical and physiological difference in the neuromuscular systems of animals and humans. The emergence of direct and indirect human somatic cell reprogramming technologies may overcome this limitation because they enable the use of disease and patient-specific cellular models as enhanced platforms for drug discovery and autologous cell-based therapy. Induced pluripotent stem cells (iPSCs) and urine-derived stem cells (USCs) are increasingly employed to recapitulate the pathophysiology of various human diseases. Recent cell-based modeling approaches utilize highly complex differentiation systems that faithfully mimic human tissue- and organ-level dysfunctions. In this review, we discuss promising cellular models, such as USC- and iPSC-based approaches, that are currently being used to model human neuromuscular and neurodegenerative diseases.

## Introduction

Understanding the mechanisms underlying the pathology of human disease is essential for drug development. Studies on fundamental principles of human disease and testing of therapeutic modalities are commonly conducted in mouse models (Partridge, 2013). The use of animal models to mimic human diseases presents challenges arising from genetic and physiological differences between humans and animals, in pathologic mechanisms and therapeutic effect. However, due to the lack of biologically-relevant human disease models, animal models represent the only available approach, which could recapitulate, a physiological and anatomical condition *in vivo*. In contrast, cellular models of human disease, which recapitulate the pathophysiology of various neuromuscular and neurodegenerative diseases, bring us closer to achieving personalized therapy for the individual patient.

It is still being debated as to which cells can be used for cell-based studies. For example, primary myoblasts are usually employed for the study of Duchenne muscular dystrophy (DMD). DMD is a devastating muscle disorder caused by frameshift mutations in the DMD gene. The responsible gene (DMD) encodes the subsarcolemmal protein, dystrophin. In DMD, various frameshift mutations in DMD prevent the full translation of dystrophin. Primary myoblasts express enough levels of dystrophin mRNA but do not express dystrophin protein. This renders them a suitable surrogate for DMD, but collecting myoblasts requires invasive muscle biopsy. Dystrophin mRNA has also been detected in lymphocytes and fibroblasts, as shown by nested real-time polymerase chain reaction (RT-PCR). However, dystrophin protein is not detected in these cells because of illegitimate dystrophin transcripts present at a very low level. Fibroblasts can be converted to myotubes by virally-mediated MyoD1 transduction. Although the transduced cells express dystrophin mRNA and protein, achieving enough expression at the protein level remains challenging. In our previous study, we overcame this issue by designing an *in vitro* assay based on MyoD1-converted fibroblasts isolated using fluorescence-activated cell sorting (FACS) to determine patient eligibility before clinical trials (Saito et al., 2010).

In the neuromuscular diseases, it is challenging to generate a disease model that faithfully represent the patient’s pathology, and there are situations in which enough efficacy and safety cannot be confirmed in clinical trials. This is in contradiction to the results in animal models. Human-induced pluripotent stem cells (iPSCs) are an attractive platform for overcoming these limitations. Therefore, patient-specific iPSCs can provide unlimited disease-relevant cells in a personalized manner. This serves as an essential resource for cell types previously considered rare or inaccessible, including skeletal and cardiac myocytes, neurons, and glia. However, there are some limitations about genome instability and epigenetic memory associated with the reprogramming of iPSC, integrity of iPSC derivatives, inherent biological and technical variability between iPSC lines and differentiated cells, and modeling of diseases that are epigenetically influenced by environmental factors or largely sporadic in etiology. Disease modeling using somatic stem cells have also been conducted as a way to solve epigenetic and environmental factors.

In this review, we discuss the current status of cellular modeling of neuromuscular and neurodegenerative diseases, and how such models can contribute towards developing precision therapies for patients with these diseases. In addition, we review a new approach to disease modeling based on urine-derived stem cells (USCs) that is used as a model for neuromuscular disease.

## Stem Cells Used for Modeling Disease

Stem cells are a valuable research tool for basic, pre-clinical, and clinical studies. Stem cells are defined by two essential characteristics; one is the ability to divide indefinitely and self-replicate, and the other is the ability to differentiate into mature cells under appropriate conditions and specific signals (Malaver-Ortega et al., 2012). According to differentiation potential, stem cells are classified as follows; totipotent, pluripotent, multipotent, oligopotent, and unipotent (Malaver-Ortega et al., 2012). Totipotent and pluripotent cells, which can differentiate into three embryonic lineages and change to any cell type, correspond to embryonic stem cells. Pluripotent stem cells can be obtained from adult somatic cells by incorporating pluripotent transcription factors into the cell’s genome. These cells are called iPSCs that have been fully reprogrammed to achieve an induced pluripotent state. Somatic stem cells, multipotent cells that can differentiate into a limited number of mature cell types, can be found among the tissues such as the brain, skeletal muscle, skin, bone marrow, blood, adipose tissue, and liver. These cells have the role of repairing damaged tissue and obtaining tissue homeostasis when tissue damage occurs. One type of somatic stem cell is a mesenchymal stem cell (MSC) that can differentiate into various mesodermal cells such as osteoblasts, chondrocytes, muscle cells, and adipocytes (Nombela-Arrieta et al., 2011). MSCs are an example of multipotent stem cells that are characterized by adherence to plastic surfaces with a wide range of proliferative potentials *in vitro* and *in vivo*. Lymphoid and myeloid cells are called oligopotent stem cells, and skeletal muscle satellite cells are examples of unipotent cells involved in muscle regeneration. These stem cells are used in cellular modeling of neuromuscular diseases, especially iPSCs are extensively applied. The generation of disease model cells with somatic cells has technical difficulties, and the number of reports on this approach is limited (Grath and Dai, 2019).

## Modeling of Muscle and Neuronal Diseases Using iPSCs

Human iPSCs are an attractive platform for overcoming the limitations of animal models in disease modeling and drug discovery. In 2006, a study from Japan showed that murine adult fibroblasts could be successfully reprogrammed by the introduction of four transcription genes including Oct3/4, Sox2, Klf4, and c-Myc *via* retroviral vectors (Takahashi and Yamanaka, 2006). In 2007, differentiated human somatic cells were reprogrammed to enter a pluripotent state allowing for the creation of patient and disease-specific stem cells (Takahashi et al., 2007). iPSCs have the capacity for self-renewal and differentiation and can also be directly generated from skin fibroblasts and blood cells of the patients as well as from other somatic cell sources. However, recently developed approaches employ lymphocytes, squamous cells, and urine-derived cells, which can be obtained in a less invasive manner. iPSCs can differentiate into almost any cell type including skeletal and cardiac myocytes, neurons, and glias. Because these cell lines are patient-specific, they are expected to recapitulate disease-specific phenotypes and elucidate the molecular mechanisms that drive neuromuscular and neurodegenerative diseases.

Obtaining tissues from patients with muscle and neuronal diseases is difficult because muscle biopsy is invasive, and brain biopsy is almost impractical, and involve risks such as pain, bleeding, infection, anesthesia-related complications, and seizures. Therefore, numerous studies are developing methods to derive myogenic and neuronal cells from iPSCs.

There are two different approaches that are commonly used to differentiate iPSCs into myogenic precursor cells (Kodaka et al., 2017). One approach involves overexpressing myogenic transcription factors, MyoD1 and Pax7, in iPSCs using integrated vectors such as lentiviruses (Maffioletti et al., 2015). This approach is highly efficient, but vector integration can lead to genotoxicity. Another method involves mimicking key signaling events such as dual modulation of the Wnt and bone morphogenetic protein signaling pathways to induce myogenesis in iPSCs (Chal et al., 2016). This direct reprogramming approach requires a month to generate robust myogenesis but avoids the need for genetic modification or cell sorting, thereby enabling abundant production of myogenic promoters for therapeutic applications.

Although optimized methods of iPSC differentiation induction have been developed (Revilla et al., 2016), the protocol for neuronal differentiation induction varies greatly depending on the desired cell type. Neuronal differentiation, development, and maturation are modeled on the progression of chemical signaling that occurs *in vivo*. The appropriate composition, concentration, and timing of the growth factor signals induce the differentiation of the target neuron. Furthermore, in order to convert to fully differentiated cells with sufficient differentiation efficiency, it is necessary to consider the environment suitable for the development and maturation of the target neuron (Engel et al., 2016). Evaluation using a disease-specific marker has been reported to have a differentiation efficiency of around 90% in cholinergic neurons (Crompton et al., 2013) and astrocytes (Krencik et al., 2011; Serio et al., 2013).

Using current protocols, iPSCs can be differentiated into cells with phenotypes resembling those of dopaminergic, glutamatergic, GABAergic, and motor neurons, those of medium spiny neurons of the striatum, and those of glial progenitors (Ross and Akimov, 2014). iPSCs are used to study various neuromuscular and neurodegenerative diseases such as spinal muscular atrophy (SMA; Sareen et al., 2012), amyotrophic lateral sclerosis (ALS; Egawa et al., 2012), Huntington’s (Kaye and Finkbeiner, 2013), Parkinson’s disease (PD; Devine et al., 2011), and Alzheimer’s disease (AD; Ooi et al., 2013).

In the study of the effects of a single genetic abnormality, it is necessary to consider the genetic background and disease-related mutations that are thoroughly permeated in the iPSCs used. Since disease-specific iPSCs exhibit a disease phenotype, their genetic background might be considered to be permissible in the phenotype. But in the genome-edited wild-type iPSCs, if a genetic background is not taken into account, the disease state is not accurately reflected (Musunuru et al., 2018). Genomic editing techniques such as CRISPR/Cas9 are currently used to minimize these variations due to genetic background. That is, use genome editing techniques to correct genetic abnormalities in patient-derived cells or introduce putative genetic abnormalities into cells derived from healthy individuals. By creating two patterns of isogenic cell pairs and comparing their phenotypes, it is thought that the underlying pathological mechanisms can be understood in more detail (Bassett, 2017).

Although iPSCs are excellent for cellular modeling in neuromuscular and neurodegenerative diseases, some limitations still remain and hinder the use of iPSC-based assays. First, collecting somatic cells for iPSC preparation may require invasive procedures. For example, the harvesting of patient-derived fibroblasts requires a skin biopsy, which is not ideal for young patients. Inducing iPSC differentiation into specific cells can be time-consuming, and may require special techniques and equipments. Differentiation efficiency of iPSCs derived from a single patient can vary among clones. Additionally, these iPSCs are heterogeneous which complicates reproducibility of directed differentiation and analyses such as high-throughput screening. Future studies will increase iPSC homogeneity which will improve differentiation efficiency. Methods used to differentiate iPSC into neuronal cells often require long-term culture during which the cells develop mature functional properties. iPSCs may require 1–2 months to differentiate into dopamine neurons, often achieving only 10–20% on the tyrosine hydroxylase index, which is used as an indicator of dopaminergic neurons (Playne and Connor, 2017). Cerebellar Purkinje cells are challenging to culture *in vitro* because of their large size, complex morphology, unique firing properties, and extended period of maturation (over 150 days; Watson et al., 2015). Patient-derived iPSCs used to model neurologic diseases need to be developed with greater efficiency than one provided by currently available methods. Additionally, several studies have shown that iPSC-derived neurons recapitulate only early-, but not late-onset, phenotypes of neurologic diseases (Nguyen et al., 2011; Patterson et al., 2012). This is primarily due to the fetal nature and immature phenotype of iPSC-derived neurons (Ho et al., 2016). The process of iPSC reprogramming, which involves an embryo-like pluripotent state, results in the loss of specific age-related characteristics (Lapasset et al., 2011). Environmental and aging-related factors also present a significant risk in late-onset neuromuscular and neurodegenerative diseases. Therefore, it is essential to establish cellular models corresponding to the fundamental features of these diseases.

## Direct Conversion of Somatic Cells Into Neurons and Muscle Cells

Previous studies have examined whether one differentiated cell type can be directly converted into another desired cell type by genetic manipulation without passing through an intermediate or pluripotent state. Such direct differentiation of mature somatic cells into other cell types was first reported by Lassar and co-workers who showed that introducing the MyoD1 transcription factor can convert fibroblasts into skeletal muscle cells (Davis et al., 1987; Tapscott et al., 1988). Thereafter, Xie et al. (2004) differentiated mature B lymphocytes into macrophages, Zhou et al. (2008) transdifferentiated exocrine cells into pancreatic endocrine cells, Ieda et al. (2010) converted fibroblasts into functional cardiomyocytes. Currently, direct cell conversion is not limited to cell types originating from the same germ layer. Human fibroblasts and hepatocytes can also be converted into functional neurons using transcription factors and/or microRNAs. Such direct cell conversion generates targeted cell types more rapidly than do iPSC-based techniques. However, methods to verify cellular identity, uniform phenotype, functionality, and safety throughout the process of transformation have not yet been established.

Several studies have shown that brain neurons and cardiomyocytes converted directly from somatic cells preserve the cellular ageing markers and possibly even the state of maturation (Qian et al., 2012; Mertens et al., 2015; Huh et al., 2016). Specifically, Tang et al. (2017) have shown that motor neurons, generated by direct reprogramming from fibroblasts can maintain the characteristics of aging donors including extensive DNA damage, loss of heterochromatin and nuclear tissue, and increased SA-β-Gal activity. Preservation of these characteristics has not been observed in iPSC-based models. Furthermore, Liu et al. (2016) demonstrated that direct reprogramming of motor neurons from fibroblasts maintain the biological age of ALS patients, showing degenerative morphology, hypoactivity, and reduced survival. These studies indicate that somatic cells converted directly into neurons without undergoing the intermediate pluripotent state may retain the age-related biochemical phenotype of the donor. As mentioned above, in neurodegenerative diseases, ageing and environmental factors influence the onset and progression of the disease state. Therefore, maintenance of the age-related phenotype is essential in the modeling of these diseases. Overall, these findings suggest that neurons obtained directly from converted somatic cells may be more appropriate than iPSCs for modeling of neurological diseases.

## Using Urine-Derived Stem Cells to Model Neuromuscular and Neurodegenerative Diseases

Cells used in *in vitro* disease research should be obtained from patients of all ages, genders, and genetic origin by procedures that are non-invasive, low-cost, and straightforward to implement (Zhang et al., 2008). Obtaining cells from urine presents a non-invasive approach, and urine is a readily available and nearly unlimited source of biological samples. In recent years, cells with stem-like characteristics have been identified in urine samples and have been recognized as useful materials in disease modeling (Falzarano and Ferlini, 2019).

## Characterization of Urine-Derived Stem Cells

USCs are progenitor cells that can self-renew and differentiate (Zhang et al., 2008). USCs can be induced to differentiate into several cell types, including endothelial cells, uroepithelial cells, smooth muscle cells, neural stem cells, and beta cells (Bharadwaj et al., 2011). USCs are thought to originate specifically from kidney glomerular parietal epithelial cells (PECs). USCs isolated from the upper urinary tract and voided USCs are similar in morphology, cell growth pattern, and differentiation potential (Zhang et al., 2014). Furthermore, USCs from a woman who received a kidney transplanted from a male donor contained a Y chromosome and exhibited normal kidney cell markers (PAX2 and PAX8; Bharadwaj et al., 2013). These findings indicate that USCs originate from the kidney and/or the upper urinary tract. USCs express specific genes, protein markers (synaptopodin and podocin) and a high percentage of CD146^+^/CD31^−^ that expressed in glomerular wall cells and podocytes (Bharadwaj et al., 2013). These markers are not detected in other cells of the urinary system, such as urinary tract epithelium and smooth muscle cells of the bladder and ureter (Bharadwaj et al., 2013). PECs have been reported to self-renew and regenerate podocytes and proximal tubule cells (Sagrinati et al., 2006; Poulsom and Little, 2009; Miesen et al., 2017). These facts strongly support that USCs are derived from PECs. USCs show high expandability that is comparable to that of other widely used stem cells such as bone marrow stem cells, blood progenitor cells, keratinocyte progenitor cells, umbilical cord stem cells, and adipose-derived stem cells (Terstegge et al., 2007; Zhang et al., 2014; Abdelalim and Emara, 2015; Guan et al., 2015). USCs express high levels of mesenchymal stem-cell markers, such as CD44, CD73, CD29, CD105, CD166, CD90, and CD13 (He et al., 2016), and pluripotent stem cell markers including POU5F1 or Oct 3/4, c-Myc, SSEA-1/4, and Klf-4 (Bharadwaj et al., 2011). USCs are multipotent and can differentiate into cells of mesodermal, endodermal and ectodermal lineage (Bharadwaj et al., 2013). USCs also show a high proliferative ability that is comparable to that of other commonly used stem cells such as bone marrow stem cells (Zhang et al., 2014), umbilical cord stem cells (Liu et al., 2018), and adipose-derived stem cells (Kang et al., 2015).

**Figure 1:**
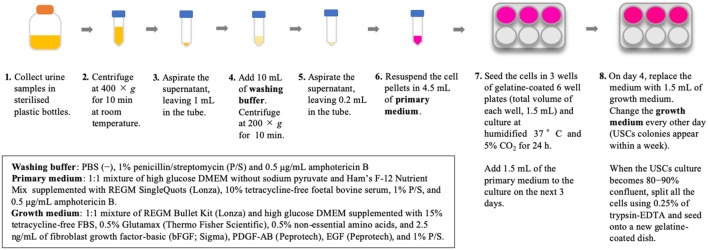
Method for isolation of urine-derived stem cells (USCs).

Isolation of USCs is straightforward and reproducible. The method for USC isolation has been described previously (Zhou et al., 2012). In our previous study, we used an existing protocol with some modifications (Takizawa et al., 2019); briefly, our methods are described in Figure 1. Urine collection is a straightforward, repeatable procedure that is non-invasive to the patient. Additionally, reducing the costs associated with cell culture presents considerable advantages. The isolation of USCs requires simple centrifugation and standard culture plates without particular substrates, which decreases isolating cost to less than US$70 per sample (Pavathuparambil Abdul Manaph et al., 2018). Conversely, iPSC reprogramming requires expertise and various types of equipment, raising the cost to more than US$120–200 per sample (Beers et al., 2015).

## Application of Urine-Derived Stem Cells in Neuromuscular Disease

Few studies have evaluated the usefulness of USCs in disease modeling and drug screening. The following summarizes the reports on the generation of cellular models for muscle and neurological diseases using USCs. One is to induce USCc into iPSC and create a cellular model (urine-derived induced pluripotent stem cells, UiPSCs), and another is to induce USC into the target cell by direct reprogramming Supplementary Table S1.

## Urine-Derived Induced Pluripotent Stem Cells

Presently, human iPSCs can be generated from various donor sources. Urine may represent an ideal source of cells for generating iPSCs. After Zhou et al. (2012) generated iPSCs from USCs, numerous studies revealed that iPSCs generated from USCs might have several advantages over iPSCs generated from other somatic cells. Induction of UiPSCs requires less time than do iPSCs derived from fibroblasts, lymphocytes, and keratinocytes (Zhou et al., 2012). Moreover, UiPSCs show a high reprogramming efficiency of 0.1%–4% (Benda et al., 2013) and generating efficiency of approximately 1.5% which is a hundredfold higher than that of fibroblasts (0.01%; Ousterout et al., 2015). It has been demonstrated that mesenchymal-to-epithelial transition (MET) is an essential early step in reprogramming fibroblasts to iPSCs, and a critical rate-limiting step during conversion (Li et al., 2010; Samavarchi-Tehrani et al., 2010). The USCs, which mainly originated from epithelial cells, do not require MET. This fact may affect their differentiation potential. Furthermore, iPSCs derived from fibroblasts may possess epigenetic memory, which is easily differentiated into a lineage related to the donor cell type, the mesoderm lineage (Kim et al., 2010, 2011). However, this tendency has not been observed in UiPSC (Shi et al., 2016).

UiPSCs have already been used in numerous studies on disease modeling (Ji et al., 2017) such as those on Type 2 long QT syndrome (Jouni et al., 2015), dilated cardiomyopathy (Lin et al., 2016), multiple endocrine neoplasia type 1 syndrome (Guo et al., 2017), hemophilia A (Jia et al., 2014), systemic lupus erythematosus (Chen et al., 2013), Down syndrome (Lee et al., 2017), SMA (Zhou et al., 2018), spinal cord injury (Liu et al., 2017), and muscular dystrophy (Afzal and Strande, 2015).

## Direct Reprogramming of Urine-Derived Stem Cells Into Myogenic Lineage

Several studies on skeletal muscle diseases indicate that USC can be induced into myogenic lineage by direct reprogramming *via* muscle transcription factor MyoD1. Falzarano et al. (2016) demonstrated that USC derived from patients with DMD retain the patient-specific DMD mutation and that USCs converted *via* MyoD1 show no dystrophin expression. Falzarano et al. (2016) additionally showed that truncated dystrophin is restored by in-framing with antisense oligonucleotides against exon 44 of *DMD*. Kim et al. (2016) demonstrated that myogenic reprogramming of urine cells derived from patients with DMD and limb-girdle muscular dystrophy (LGMD) type 2 could recapitulate the disease phenotype. They additionally showed that USC genomes could be edited using CRISPR/Cas9.

Recently, we developed a novel MyoD1-converted, urine-derived cell to *in vitro* model of the pathological processes of muscle cells affected by DMD (Takizawa et al., 2019). In that study, we showed that 3-deazaneplanocin A hydrochloride (DZNep) promotes the differentiation of USCs into myotubes. DZNep-treated USCs, differentiated *via* MyoD1, are excellent *in vitro* models of muscle cells affected by DMD. Moreover, this system, which is based on urine-derived cells obtained from patients with DMD, can be successfully used to evaluate exon skipping therapy using antisense oligonucleotide for DMD. This newly-established *in vitro* assay will be used in a wide range of studies regardless of age, sex, and muscular disease type of patients. Direct reprogramming of USCs could potentially be used to study the pathophysiology of various diseases, and to diagnose and develop novel therapies for patients with these conditions.

## Direct Reprogramming of Urine-Derived Stem Cells Into Neuronal Lineage

Urine-derived cells can be differentiated into neural-lineage cells by culture in neural induction medium supplemented with basic fibroblast growth factor (Bharadwaj et al., 2013; Guan et al., 2014; Zhang et al., 2016). However, few studies have examined the direct differentiation of neuronal cells from urine-derived cells. Wang et al. (2013) developed integration-free and expandable human neural progenitor cells which can self-renew and differentiate into multi-functional neuronal subtypes and glial cells *in vitro*. Several groups reported that approximately 40% of the induced cells express several neural markers and show neurogenic extensions and processes (Bharadwaj et al., 2013; Guan et al., 2014). USCs treated with growth factors and cultured on laminin-treated plates readily convert into immature neuronal cells (Guan et al., 2014; Kim et al., 2018). Human urinary cells can be converted into neural stem cells by a non-integration-free method using small molecules, and it takes less time than through iPSCs (Cheng et al., 2014). The induced neural progenitor cells can then be converted into astrocytes, oligodendrocytes, and neurons, and may play an essential role in identifying and developing safe and effective therapies for patients with neurodegenerative conditions.

## Future Aspects of Using Urine-Derived Stem Cells as Cellular Models of Human Disease

**Figure 2:**
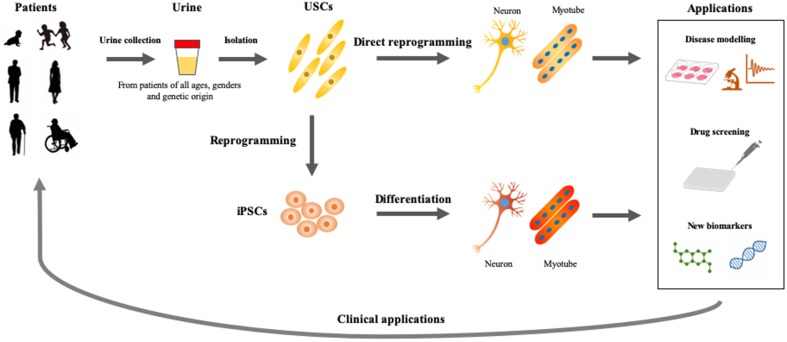
Applications of USCs as cellular models of human diseases. Urine represents an ideal material, which could be obtained from patients of all ages and genders by non-invasive and straightforward procedures. USCs- and induced pluripotent stem cells (iPSCs)-based disease modelings would be useful for basic and applied research, which accelerate the development of personalized medicine.

As discussed previously, USCs also present an advantageous *in vitro* model to study disease mechanisms, identify new biomarkers, evaluate therapeutic approaches, and screen drugs (Figure 2). USCs can be obtained reliably and non-invasively in a short period of time and cultured at low cost. This newly-established *in vitro* assay can, therefore, be adapted for various studies and platforms. Direct reprogramming is an efficient and economical approach because it can be used to generate patient-specific cell lineages without the presence of iPSC intermediates. USCs could show superior differentiation, and can, therefore, be used to delineate the mechanisms of common and rare genetic diseases and to screen drugs for the treatment of these conditions. However, the directed differentiation efficiency of USCs used to produce the mature target-cell types, needs to be optimized. Models recapitulating neurological diseases *in vitro* need to possess not only the gene and protein expression of neuron-surface markers but also characteristics of functional maturation such as synapses and cellular homeostasis. Neurons obtained directly from reprogrammed USCs must be evaluated *via* genetic, biological, and electrophysiological assessment. Future differentiation of neural cells from USCs will be inspired by the previous studies on iPSC reprogramming and other somatic cells direct reprogramming. USCs may play a complementary role in developing methods for rapid and efficient creation of iPSCs, and can be used to directly generate relevant cells for *in vitro* disease models.

There is some essential and critical issue about USCs that there is limited understanding about the biological characteristics and the ability to differentiate into other cell lines of USC as mentioned above. The neurons differentiated from USCs are not enough to determine neuronal model cells by functional evaluation, and cannot clarify the pathological mechanism. However, as mentioned above, there is a lot of potentials for USC to become a new platform, making it possible to supplement the issues of iPSC technology and to approach pathophysiology from a new aspect.

The USCs platform is still in the infancy stage and expected that this technology will be further developed and adapted by a wider research community. We believe that USCs will play an essential role in the study of diseases and drug screening. Future studies should develop more advanced USC-based differentiation systems, which will faithfully recapitulate human tissue-level and organ-level dysfunction (Rowe and Daley, 2019).

## Conclusions

The difficulties present in obtaining brain and muscular tissue, and lack of adequate preclinical models with high predictive and translational power, pose limitations in the study of neuromuscular diseases and also in developing effective drugs for patients with these disorders. Currently, advances in human iPSC-based technologies are clearly helping to overcome the limitations. Additionally, USC-based modeling will provide valuable information for establishing a diagnosis and providing effective treatment options, although USCs have not been extensively investigated in disease modeling. It should be a critical question of whether the iPSCs and USCs will be able to mimic the complexity of the human neuromuscular system or not.

Application of iPSCs and USCs will be useful for predicting drug response and assessing environmental disease triggers in neuromuscular and neurodegenerative diseases. The development of USCs- and iPSCs-based technology provides a new platform in the field of disease modeling and works in complementary ways, it is expected to benefit research and clinical applications in personalized medicine.

## Author Contributions

MS and YA wrote the initial draft of the manuscript. HT assisted in the preparation and wrote the manuscript. BT, FS, and AN have contributed critically and reviewed the manuscript.

## Conflict of Interest

The authors declare that the research was conducted in the absence of any commercial or financial relationships that could be construed as a potential conflict of interest.
