# Supplementary material for: Application of Urine-Derived Stem Cells to Cellular Modeling in Neuromuscular and Neurodegenerative Diseases
Source: Front Mol Neurosci. 2019 Dec 5;12:297. doi: 10.3389/fnmol.2019.00297 (PMC6915080; doi:10.3389/fnmol.2019.00297)
Supplement: Supplementary file 1 [file Table_1.DOCX]

**Table. Neuronal and muscular differentiation from urine-derived stem cells**

| Neuronal differentiation | | | | | | | | |
| --- | --- | --- | --- | --- | --- | --- | --- | --- |
|  | Induced cell type | Reprogramming factors | Delivery methods of factor | Markers | Culture time  (after differentiation) | Differentiation efficiency | Functional test | References |
| Direct reprogramming | Neuron | Ascl1, Brn2, NeuroD, c-Myc, Myt1l | Retrovirus | TUBB3, MAP2, SYP, GABA, VGLUT1 | Day 25 | 1.55%  (β-Tubulin III positive) | Action potential (current-clamp whole-cell configuration) | (Zhang et al., 2016) |
|  | Neuron | RA, B27, nonessential amino acid, PDGF-BB, Laminin | Chemicals | TUBB3, NES, MAP2, NF-M, NeuN | Day 14 | N.A. | - | (Kim et al., 2018) |
|  | Neuron | NeuroCult NS-A differentiation Kit (StemCell) | Chemicals | TUBB3 | Day 7 | N.A. | - | (Kang et al., 2015) |
|  | Neural progenitor cell | hEGF, bFGF, B27, nonessential amino acid, insulin-transferrin-selenite | Chemicals | TUBB3, NES, SOX2 | Day 12 | N.A. | - | (Guan et al., 2014) |
|  | Neural progenitor cell | VPA, CHIR99021, REPSOX  in Hypoxia (5% O_2_) | Chemicals | TUBB3, MAP2, GFAP | Day 20 | >60%  (Nestin and Sox2 positive) | - | (Cheng et al., 2014) |
| Indirect reprogramming through UiPSCs | Neuron | APEL media (Stem Cell Technologies), N2B27, RA | Chemicals | TUBB3 | Day 12 | N.A. | - | (Gaignerie et al., 2018) |
|  | Glutamatergic neuron | N2B27, LDN193189, SB432542, Cyclopamine, bFGF | Chemicals | TUBB3, TBR1, GLUL | Day 20 | N.A. | Action potential (current-clamp whole-cell configuration) | (Lee et al., 2017) |
|  | Motor neuron | CHIR, SB, DMH1, RA, Pur, GDNF, BDNF, IGF, CpdE | Chemicals | TUBB3, NES, OLIG2, PAX6, HB9, ISL1, CHAT | Day 26 | 79%  (HB9 positive) | - | (Yi et al., 2018) |
| Muscular differentiation | | | | | | | | |
|  | Disease modelling | Reprogramming factors | Delivery methods of factor | Markers | Culture time  (after differentiation) | Differentiation efficiency | Application | Reference |
| Direct reprogramming | DMD, LGMD2 | MyoD1 | Lentivirus | MYOG, MYH3 | Day 28 | >80% | Gene editing using CRISPR/Cas9 technology | (Kim et al., 2016) |
|  | DMD | MyoD1 | Adenovirus | MYOD1, MYH3, DMD | Day 3 | N.A. | Exon 44 skipping with antisense oligonucleotides | (Falzarano et al., 2016) |
|  | DMD | MyoD1 | Retrovirus | MYH3, DMD, MYOG | Day 14 | N.A. | Exon 44, 50, 51 or 55 skipping with antisense oligonucleotides | (Takizawa et al., 2019) |
|  | DM1, DM2 | MyoD1 | Lentivirus | MYOD1, ACTN, DMD | Day 28 | 70% | - | (Kim et al., 2019) |
|  | - | PDGF-BB, HGF | Microbeads containing growth factors injected | MYOD1, MYF-5, DES | Day 28  (after implantation) | N.A. | - | (Liu et al., 2013) |
|  | - | Conditioned medium obtained from skeletal muscle cell culture | - | MYOD1 | Day 14 | N.A. | - | (Kang et al., 2015) |
|  | - | Conditioned medium obtained from skeletal muscle cell culture | - | MYOD1, MYOG, MYF-5, MYH3 | Day 14~1 month | 50%–60% | - | (Bharadwaj et al., 2013) |
|  | - | - | USCs implanted into mouse TA | MYF-5, MYOD1, MYH3, DES | 1 month | N.A. | - | (Chen et al., 2017) |
